# Application and Outcomes of Coronary Atherectomy in Acute Coronary Syndrome: A Report From the J-PCI Registry

**DOI:** 10.1016/j.jscai.2025.103622

**Published:** 2025-05-13

**Authors:** Tadao Aikawa, Yuichiro Mori, Shun Kohsaka, Yuya Matsue, Toshiki Kuno, Kyohei Yamaji, Dai Ozaki, Takashi Tokano, Ken Kozuma, Tohru Minamino

**Affiliations:** a Department of Cardiovascular Biology and Medicine, Juntendo University Graduate School of Medicine, Tokyo, Japan; b Department of Human Health Sciences, Kyoto University, Kyoto, Japan; c Department of Cardiology, Keio University School of Medicine, Tokyo, Japan; d Cardiology Division, Massachusetts General Hospital, Harvard Medical School, Boston, Massachusetts; e Division of Cardiology, Montefiore Medical Center, Albert Einstein College of Medicine, New York, New York; f Department of Cardiovascular Medicine, Kyoto University Graduate School of Medicine, Kyoto, Japan; g Department of Cardiology, Juntendo University Urayasu Hospital, Urayasu, Japan; h Division of Cardiology, Department of Internal Medicine, Teikyo University School of Medicine, Tokyo, Japan

**Keywords:** acute coronary syndrome, coronary atherectomy, in-hospital mortality, orbital atherectomy, rotational atherectomy

## Abstract

**Background:**

Acute coronary syndrome (ACS) is considered a relative contraindication to coronary atherectomy; however, expert consensus documents do not preclude its use for ACS. We investigated the temporal trends and hospital variability in the utilization and outcomes of coronary atherectomy among patients with ACS undergoing percutaneous coronary intervention (PCI) using data from a Japanese nationwide registry.

**Methods:**

First, we analyzed the temporal trend in the use of rotational atherectomy (RA) and orbital atherectomy during PCI for patients with ACS between 2014 and 2022 (822,237 PCI across 1269 hospitals). Next, we assessed the outcomes of the patients who underwent RA for ACS between 2019 and 2022 (7421 PCI across 662 hospitals). The primary outcome was in-hospital mortality after PCI. We also evaluated the effects of PCI volumes and the 2020 policy change in Japan that allowed operators to perform coronary atherectomy at low PCI volume hospitals (<200 PCI/y) without on-site surgical backup on outcomes.

**Results:**

The overall usage rates of RA and orbital atherectomy for ACS were low, at 2.0% and 0.8%, respectively. RA for ACS was never performed at 581 hospitals (46%). After adjusting for the baseline characteristic, in-hospital mortality after RA for ACS was not significantly associated with hospital PCI volumes (odds ratio, 0.94; 95% CI, 0.65-1.36; *P* = .73 for highest vs lowest tertiles) or the initiation of coronary atherectomy after the policy change (odds ratio, 0.99; 95% CI, 0.63-1.51; *P* = .95).

**Conclusions:**

Coronary atherectomy for ACS is infrequently performed in Japan. PCI volume and on-site surgical backup were not significantly associated with in-hospital mortality after coronary atherectomy for ACS.

## Introduction

The strategy of percutaneous coronary intervention (PCI) for severe coronary calcification has been improved substantially, primarily owing to advances in coronary atherectomy devices.^1^ Specifically, rotational atherectomy (RA) and orbital atherectomy (OA) can debulk coronary calcification, enhancing stent delivery and procedural success.^1^, ^2^, ^3^, ^4^ Consequently, the ACC/AHA/SCAI guideline recommends plaque modification with RA in patients with heavily calcified lesions as class 2a and OA as class 2b,^5^ whereas the current Japanese guideline recommends that with RA as class 2a.^6^ The use of these atherectomy devices ranges from 1% to 3% of PCI in contemporary practice.^3^^,^^7^^,^^8^

In contrast to chronic coronary syndrome, the role of atherectomy devices in acute coronary syndrome (ACS) remains controversial. Owing to the occlusive thrombus formation in ACS,^9^ there is concern that using atherectomy devices may increase the risk of slow flow or no reflow and distal embolization.^1^ Consequently, ACS is considered a relative contraindication to coronary atherectomy.^10^ However, RA for non–ST-segment elevation (NSTE)-ACS is feasible and may be safely performed without increasing midterm mortality or procedure-related complications compared to that for chronic coronary syndrome.^11^, ^12^, ^13^, ^14^, ^15^ To date, European^1^ and Japanese expert consensus documents^2^ on coronary atherectomy do not preclude the use of atherectomy devices during primary PCI for ACS. Notably, approximately 30% of patients with ACS have moderate to severe coronary calcification in the culprit lesion,^16^ potentially requiring coronary atherectomy during primary PCI.^17^ Hence, a large-scale study on outcomes and their determinants after coronary atherectomy in patients with unselected ACS is still required.

In Japan, until April 2020, PCI operators were not permitted to use coronary atherectomy devices at low-volume hospitals (<200 PCI/y) or without the presence of full-time cardiovascular surgeons in their facility (ie, on-site surgical backup). This facility criterion was revised in April 2020, allowing operators to perform coronary atherectomy at low-volume hospitals without on-site surgical backup,^2^ potentially enhancing the use of coronary atherectomy for ACS at these newly accredited hospitals. Understanding these trends and hospital variability is crucial to promoting the appropriate use of coronary atherectomy devices in patients with ACS.

This study aimed to investigate the temporal trends and hospital variability in utilization and outcomes of coronary atherectomy for ACS concerning the policy change regarding coronary atherectomy, using a nationwide PCI registry.

## Methods

The availability of data supporting the results of this study is limited because they were used for the current study under license from the Japanese Association of Cardiovascular Intervention and Therapeutics (CVIT) and are not publicly available. However, the data are available from the authors upon request and with permission from the CVIT.

### National PCI data registry (J-PCI registry) and study population

The J-PCI registry is a prospective Japanese nationwide multicenter registry of PCI managed by the CVIT. It collects data on clinical characteristics and in-hospital outcomes of patients undergoing PCI at over 1000 hospitals in Japan (>90% of all PCI). The design of the J-PCI registry and the definition of variables have been described in detail previously.^18^, ^19^, ^20^ The study protocol of the J-PCI registry was approved by the Institutional Review Board Committee of the Network for Promotion of Clinical Studies (a specified nonprofit organization affiliated with Osaka University Graduate School of Medicine [Osaka, Japan]) in compliance with the Declaration of Helsinki. Written informed consent was waived because of the retrospective and observational nature of the study.

**Figure 1:**
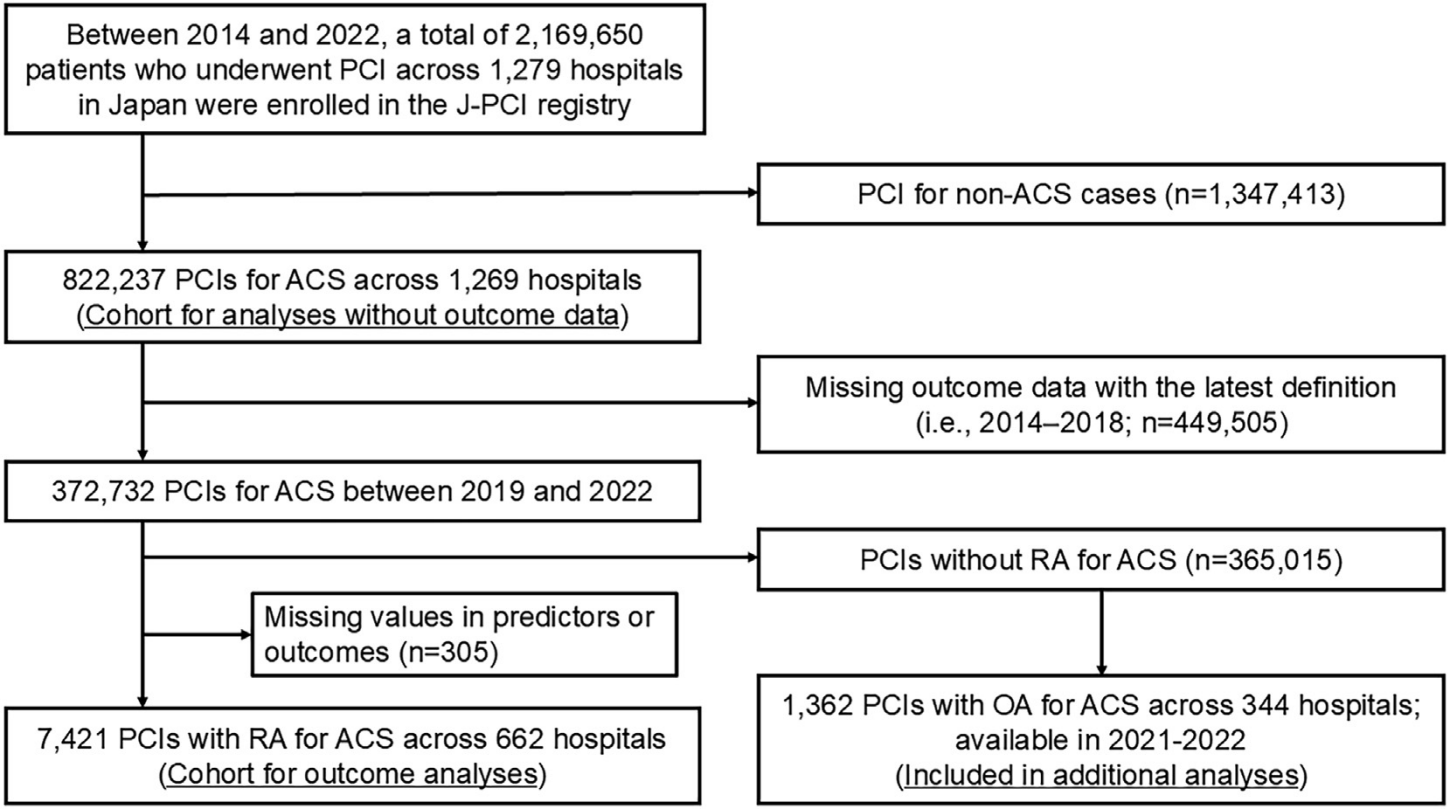
**Flow diagram for identification of the study population.** ACS, acute coronary syndrome; OA, orbital atherectomy; PCI, percutaneous coronary intervention; RA, rotational atherectomy.

For the present study, we analyzed the data of patients registered in the J-PCI registry between January 2014 and December 2022 (n = 2,169,650). After excluding PCI for non-ACS (62.1%; n = 1,347,413), 822,237 (37.9%) PCI for ACS across 1269 hospitals were included to assess the temporal trends in the use of coronary atherectomy (RA and OA) during PCI for ACS cases in Japan. Next, to analyze the clinical outcomes after PCI with RA in patients with ACS, we focused on data collected between January 2019 and December 2022 (n = 372,732), excluding data from January 2014 to December 2018 (n = 449,505). This exclusion was due to updates in outcome definitions within the J-PCI registry, which were last revised in January 2019. The updated outcome definitions are available online.^18^^,^^21^ The definitions of the ACS categories, including ST-segment elevation myocardial infarction (STEMI), NSTEMI, and UA, have been previously published^18^ and are shown in Supplemental Methods. We also excluded PCI without RA in patients with ACS (n = 365,015) and those with missing values for clinical characteristics or outcomes (n = 305). Finally, the remaining 7421 PCI with RA for ACS across 662 hospitals were included in the cohort for outcome analyses (Figure 1).

In the J-PCI registry, data on OA were available from 2021. Therefore, we performed an exploratory outcome analysis using the data on PCI with OA for ACS between 2021 and 2022 (n = 1362) across 344 hospitals to investigate the impact of RA and/or OA on short-term mortality and complications after PCI for ACS cases.

### Clinical outcomes

The primary outcome was in-hospital mortality, defined as the rate of death before hospital discharge or within 30 days after PCI, in cases where hospitalization exceeded 30 days after PCI. Secondary outcomes included in-hospital bleeding requiring blood transfusion, and a composite of in-hospital death and periprocedural complications, including PCI-related myocardial infarction, stent thrombosis (“definite” as defined by the Academic Research Consortium),^22^ cardiac tamponade, cardiogenic shock requiring mechanical and/or inotropic support, emergency surgery, and bleeding requiring blood transfusion, which was defined as any complications.

### Statistical analyses

Continuous variables were presented as the mean ± standard deviation. Categorical variables were presented as frequencies and percentages. Differences between the groups were assessed using a 1-way analysis of variance for continuous variables and the χ^2^ test for categorical variables.

In Japan, PCI operators were not permitted to use coronary atherectomy devices at low-volume hospitals (<200 PCI/y) or without on-site surgical backup until April 2020. This facility criteria were revised in April 2020, which allowed operators to perform coronary atherectomy at low-volume hospitals without on-site surgical backup after undergoing device-specific training on coronary atherectomy.^2^ Therefore, patient, hospital, and procedural characteristics and in-hospital outcomes after RA were presented according to the calendar period of performing RA: before (2019) and after (2020-2022) the 2020 policy change on coronary atherectomy in Japan.

A multivariable logistic regression analysis was performed to identify characteristics associated with the primary and secondary outcomes for 2 groups: (1) RA cases only, and (2) RA or OA cases. Patient demographics and comorbidities, preprocedural, procedural, and hospital characteristics listed in Table 1 were all included as covariates in the multivariable analysis. Annual hospital PCI volume was categorized into tertiles and compared with the lowest tertile. As an exploratory analysis, we assessed the safety of RA for ACS at hospitals without on-site surgical backup that launched coronary atherectomy after the 2020 policy change. Associations between outcomes and covariates were presented as adjusted odds ratios with 95% confidence intervals. Statistical analysis was performed using R version 4.1.2 (R Foundation for Statistical Computing). For all tests, a 2-sided *P* < .05 was considered statistically significant.

**Table 1:** Temporal changes in baseline characteristics of ACS patients undergoing rotational atherectomy.

Characteristics	Before the policy change	After the policy change			*P* value
	2019 (n = 1723)	2020 (n = 1703)	2021 (n = 1995)	2022 (n = 2000)	
Demographics					
Mean age, y	75.6 ± 9.9	75.6 ± 9.5	75.9 ± 9.7	76.2 ± 9.8	.14
Female sex	557 (32.3)	546 (32.1)	651 (32.6)	667 (33.4)	.85
History					
Hypertension	1450 (84.2)	1446 (84.9)	1689 (84.7)	1671 (83.6)	.67
Diabetes	942 (54.7)	976 (57.3)	1138 (57.0)	1140 (57.0)	.36
Hyperlipidemia	1121 (65.1)	1157 (67.9)	1333 (66.8)	1381 (69.0)	.065
Current or recent smoker (within 1 y)	401 (23.3)	384 (22.5)	462 (23.2)	444 (22.2)	.84
Chronic kidney disease	714 (41.4)	707 (41.5)	887 (44.5)	897 (44.9)	.054
Dialysis	416 (24.1)	426 (25.0)	486 (24.4)	457 (22.9)	.47
Chronic lung disease	58 (3.4)	50 (2.9)	88 (4.4)	68 (3.4)	.090
Peripheral arterial disease	215 (12.5)	242 (14.2)	251 (12.6)	264 (13.2)	.40
Prior PCI	783 (45.4)	797 (46.8)	934 (46.8)	884 (44.2)	.30
Prior CABG	150 (8.7)	147 (8.6)	185 (9.3)	159 (8.0)	.53
Prior myocardial infarction	391 (22.7)	465 (27.3)	513 (25.7)	514 (25.7)	.018
Preprocedural characteristics					
STEMI	268 (15.6)	267 (15.7)	347 (17.4)	326 (16.3)	.40
Cardiac arrest within 24 hours	61 (3.5)	44 (2.6)	62 (3.1)	65 (3.2)	.43
Cardiogenic shock within 24 hours	134 (7.8)	136 (8.0)	177 (8.9)	156 (7.8)	.55
Acute heart failure within 24 hours	177 (10.3)	187 (11.0)	249 (12.5)	231 (11.6)	.19
Preprocedural antiplatelet therapy	1626 (94.4)	1627 (95.5)	1897 (95.1)	1912 (95.6)	.29
Preprocedural asprin	1569 (91.1)	1569 (92.1)	1800 (90.2)	1812 (90.6)	.21
Preprocedural clopidogrel	645 (37.4)	657 (38.6)	655 (32.8)	574 (28.7)	<.001
Preprocedural potent P2Y12 inhibitors (ticagrelor or prasugrel)	869 (50.4)	825 (48.4)	1106 (55.4)	1196 (59.8)	<.001
Preprocedural anticoagulants	113 (6.6)	136 (8.0)	165 (8.3)	152 (7.6)	.23
Procedural characteristics					
Arterial access site					<.001
Femoral	821 (47.6)	751 (44.1)	860 (43.1)	813 (40.6)	–
Radial	777 (45.1)	827 (48.6)	1011 (50.7)	1062 (53.1)	–
Others	125 (7.3)	125 (7.3)	124 (6.2)	125 (6.2)	–
Three-vessel disease	394 (22.9)	403 (23.7)	507 (25.4)	490 (24.5)	.30
LMT or proximal LAD disease	724 (42.0)	737 (43.3)	893 (44.8)	900 (45.0)	.23
Drug-eluting stent use	1373 (79.7)	1334 (78.3)	1511 (75.7)	1461 (73.0)	<.001
Drug-coated balloon use	367 (21.3)	434 (25.5)	562 (28.2)	644 (32.2)	<.001
Mechanical circulatory support during PCI	241 (14.0)	251 (14.7)	358 (17.9)	384 (19.2)	<.001
Hospital characteristics					
Hospitals launching coronary atherectomy after the device policy change in 2020	0 (0)	113 (6.6)	283 (14.2)	394 (19.7)	<.001
Mean annual PCI volume	541.9 (454.5)	443.3 (285.7)	440.3 (317.5)	442.4 (377.7)	<.001
Annual PCI volume tertile					<.001
Low	187 (10.9)	271 (15.9)	425 (21.3)	419 (21.0)	–
Middle	557 (32.3)	589 (34.6)	617 (30.9)	703 (35.1)	–
High	979 (56.8)	843 (49.5)	953 (47.8)	878 (43.9)	–

Values are mean ± SD or n (%).

ACS, acute coronary syndrome; CABG, coronary artery bypass grafting; LAD, left anterior descending artery; LMT, left main trunk; PCI, percutaneous coronary intervention; STEMI, ST-segment elevation myocardial infarction.

## Results

### Temporal trends and hospital variations in the use of coronary atherectomy for ACS

**Figure 2:**
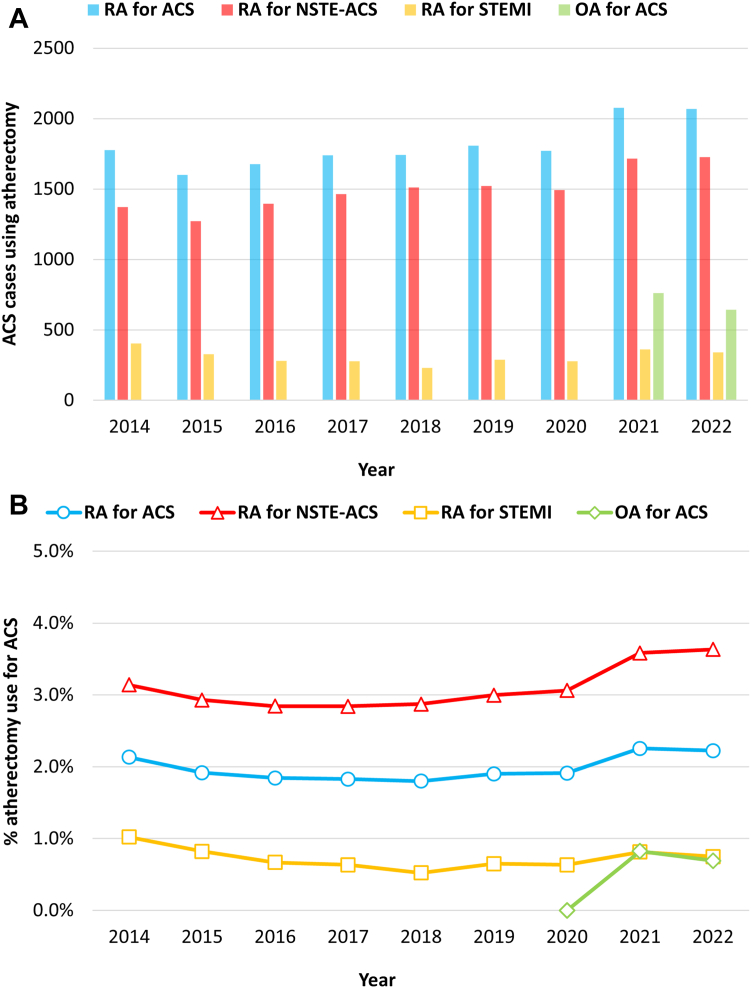
**Trends in the use of rotational atherectomy (RA) and orbital atherectomy (OA) for acute coronary syndrome (ACS) according to clinical presentation based on the numbers (A) and percentage (B) of percutaneous coronary intervention cases.** NSTE-ACS, non–ST-segment elevation acute coronary syndrome; STEMI, ST-segment elevation myocardial infarction.

Among patients with ACS undergoing PCI, trends in the use of RA according to clinical presentation are shown in Figure 2. Between January 2014 and December 2022, the rate of PCI with RA for ACS was low at 2.0% in the overall cohort (16,264/822,237 PCI) and remained low at 2.1% even after the 2020 policy change on coronary atherectomy in Japan. The use of RA for non–ST-segment elevation acute coronary syndrome (NSTE-ACS) increased from 2.9% to 3.4% after the policy change. The rate of RA for STEMI did not increase during the overall study period (≤1.0 % of PCI for ACS). The rate of PCI with OA for ACS was also low at 0.8% in 2021-2022 (1404/185,141 PCI).

**Figure 3:**
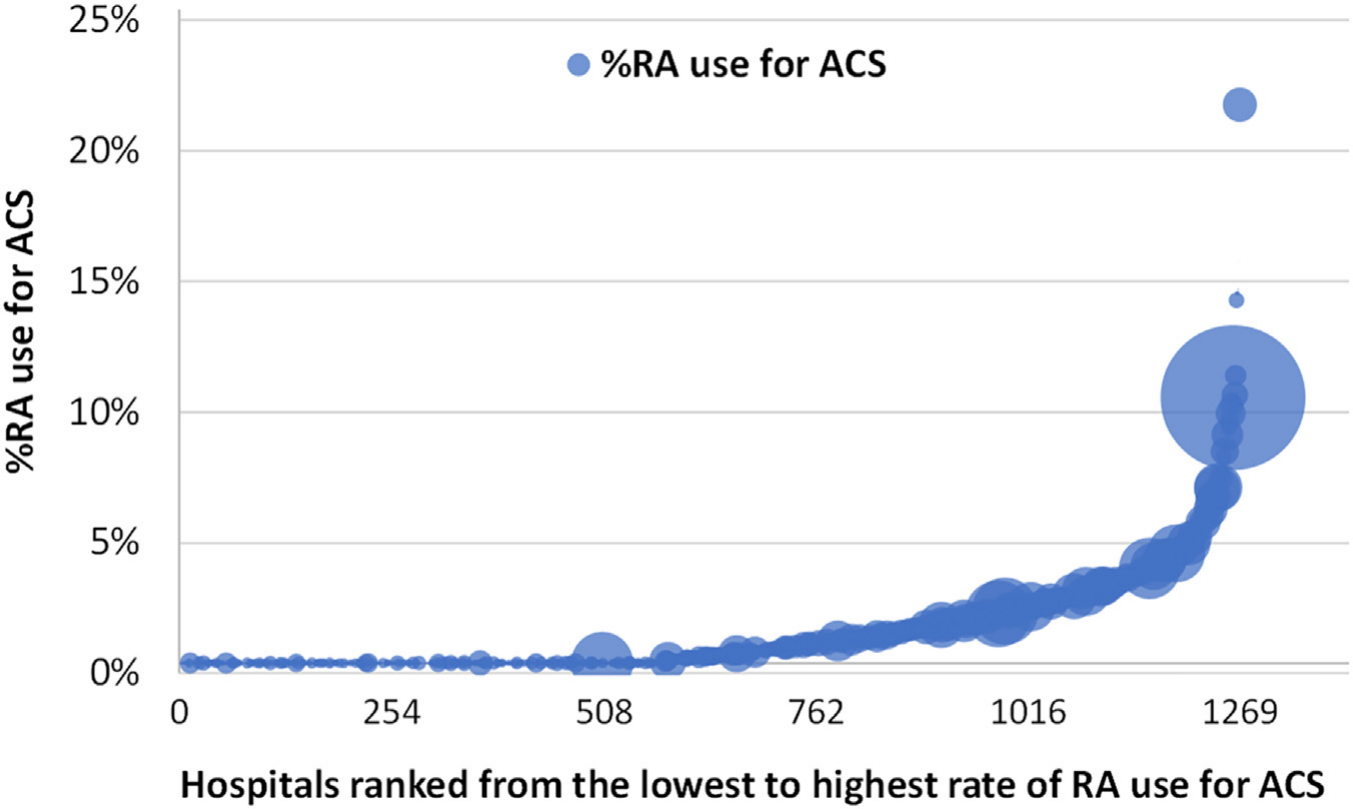
**Bubble chart of the rates of rotational atherectomy (RA) use for acute coronary syndrome (ACS) across 1269 Japanese hospitals between 2014 and 2022.** The number of percutaneous coronary interventions for ACS performed in each hospital between 2014 and 2022 is expressed by the diameter of the bubble.

RA for ACS was not performed at 581 of 1269 hospitals (46%) between 2014 and 2022. Significant variation was observed in the rate of PCI with RA for ACS among hospitals that performed at least 1 RA for ACS between 2014 and 2022 (median, 1.5%; interquartile range [IQR], 0.7%-2.8%; maximum, 21.4% [446/2087 PCI for ACS]; Figure 3).

### Population characteristics of patients with ACS undergoing RA

Baseline characteristics of patients with ACS undergoing RA between 2019 and 2022 are shown in Table 1. Patients with ACS undergoing RA were older, predominantly male, and had hypertension, diabetes, and hyperlipidemia. According to the clinical presentation before PCI, 16.3% (1208/7421 PCI) of patients with ACS presented with STEMI. The use of radial artery access increased from 45.1% in 2019 to 53.1% in 2022. The use of drug-eluting stents was common but decreased from 79.7% in 2019 to 73.0% in 2022. The use of drug-coated balloons increased from 21.3% in 2019 to 32.2% in 2022 (Supplemental Figure S1).

Coronary atherectomy for ACS increased notably in hospitals that began performing coronary atherectomy after the 2020 policy change, reaching a rate of 19.7% in 2022. Consequently, the proportion of RA cases performed at hospitals in the lowest tertile of annual PCI volume increased substantially (10.9% in 2019 to 21.0% in 2022).

### In-hospital outcomes after RA for ACS

In-hospital outcomes of patients with ACS undergoing RA are shown in Table 2. The crude in-hospital mortality rate did not significantly change over time (4.6% to 5.8%, *P* = .34). Similarly, PCI-related complications such as PCI-related myocardial infarction, cardiac tamponade, cardiogenic shock requiring mechanical and/or inotropic support, stent thrombosis, emergency surgery requirement, and bleeding requiring blood transfusion did not show significant changes over time (trend *P* > .05 for all).

**Table 2:** In-hospital outcomes after rotational atherectomy for acute coronary syndrome.

Outcomes	Before the policy change	After the policy change			*P* value
	2019 (n = 1723)	2020 (n = 1703)	2021 (n = 1995)	2022 (n = 2000)	
In-hospital death	79 (4.6)	80 (4.7)	105 (5.3)	115 (5.8)	.34
Complications					
PCI-related myocardial infarction	27 (1.6)	26 (1.5)	34 (1.7)	33 (1.7)	.97
Cardiac tamponade	11 (0.6)	10 (0.6)	11 (0.6)	10 (0.5)	.95
Cardiogenic shock requiring mechanical and/or inotropic support	39 (2.3)	64 (3.8)	64 (3.2)	61 (3.0)	.086
Stent thrombosis	3 (0.2)	4 (0.2)	1 (0.1)	3 (0.1)	.52
Requirement for emergency surgery	6 (0.3)	1 (0.1)	4 (0.2)	3 (0.1)	.26
Bleeding requiring blood transfusion	12 (0.7)	20 (1.2)	29 (1.5)	23 (1.1)	.19
Access site bleeding	24 (1.4)	37 (2.2)	44 (2.2)	35 (1.8)	.23
Nonaccess site bleeding	12 (0.7)	17 (1.0)	15 (0.8)	13 (0.7)	.64
In-hospital death or any complication	147 (8.5)	174 (10.2)	206 (10.3)	211 (10.5)	.16

Values are n (%).

PCI, percutaneous coronary intervention.

Odds ratios for in-hospital death adjusted for baseline characteristics in patients with ACS undergoing RA are shown in Table 3. Increasing the complexity of baseline characteristics, such as advanced age, diabetes, chronic kidney disease, dialysis, peripheral arterial disease, prior coronary artery bypass grafting, STEMI, cardiac arrest within 24 hours, cardiogenic shock within 24 hours, 3-vessel disease, and mechanical circulatory support during PCI, were significantly associated with increased in-hospital mortality after RA in patients with ACS (*P* < .05 for all). The transradial approach was significantly associated with a lower odds ratio of in-hospital death after RA for ACS than the transfemoral approach (adjusted odds ratio, 0.72; 95% CI, 0.54-0.96, *P* = .025). Annual hospital PCI volume was not significantly associated with in-hospital death after RA for ACS (odds ratio, 0.90; 95% CI, 0.65-1.27; *P* = .54 for the highest tertiles vs lowest tertiles). In both NSTE-ACS or STEMI patient groups, advanced age and critical preoperative conditions, including cardiac arrest within 24 hours, cardiogenic shock within 24 hours, and mechanical circulatory support during PCI, were significantly associated with increased in-hospital mortality after RA (*P* < .05 for all).

**Table 3:** Adjusted odds ratios of in-hospital death after rotational atherectomy for acute coronary syndrome.

Variables	All RA cases		RA for NSTE-ACS		RA for STEMI	
	Odds ratio (95% CI)	*P* value	Odds ratio (95% CI)	*P* value	Odds ratio (95% CI)	*P* value
Age, per 1 y	1.06 (1.05-1.08)	<.001	1.08 (1.06-1.10)	<.001	1.04 (1.01-1.06)	.004
Female sex	1.12 (0.86-1.45)	.41	1.18 (0.85-1.62)	.32	1.16 (0.73-1.84)	.53
Hypertension	0.79 (0.59-1.08)	.14	0.70 (0.49-1.04)	.068	0.98 (0.58-1.69)	.94
Diabetes	1.42 (1.11-1.83)	.006	1.30 (0.95-1.77)	.10	1.61 (1.04-2.52)	.033
Hyperlipidemia	0.61 (0.47-0.78)	<.001	0.53 (0.39-0.72)	<.001	0.81 (0.53-1.25)	.35
Current/recent smoker (within 1 year)	0.97 (0.71-1.32)	.87	1.10 (0.74-1.60)	.63	0.68 (0.39-1.16)	.17
Chronic kidney disease	1.54 (1.17-2.03)	.002	1.56 (1.10-2.21)	.012	1.48 (0.92-2.36)	.11
Dialysis	1.56 (1.12-2.17)	.008	1.81 (1.22-2.67)	.003	1.16 (0.61-2.19)	.65
Chronic lung disease	1.24 (0.69-2.11)	.44	1.35 (0.66-2.54)	.37	1.25 (0.42-3.18)	.66
Peripheral arterial disease	1.49 (1.09-2.03)	.012	1.89 (1.32-2.68)	<.001	0.73 (0.36-1.42)	.37
Prior PCI	0.73 (0.55-0.97)	.032	0.75 (0.53-1.05)	.094	0.85 (0.48-1.47)	.57
Prior CABG	1.88 (1.29-2.71)	.001	2.06 (1.35-3.09)	.001	1.87 (0.72-4.60)	.18
Prior myocardial infarction	1.23 (0.91-1.66)	.18	1.66 (1.17-2.35)	.004	0.48 (0.24-0.91)	.029
STEMI	1.89 (1.44-2.46)	<.001	–	–	–	–
Cardiac arrest within 24 hours	3.39 (2.32-4.94)	<.001	3.42 (2.01-5.78)	<.001	3.38 (1.94-5.92)	<.001
Cardiogenic shock within 24 hours	3.13 (2.31-4.24)	<.001	3.58 (2.43-5.25)	<.001	2.54 (1.53-4.18)	<.001
Preprocedural potent P2Y12 inhibitors (ticagrelor or prasugrel)	1.19 (0.93-1.51)	.17	1.34 (1.00-1.80)	0.053	0.90 (0.59-1.40)	.65
Preprocedural anticoagulants	0.95 (0.61-1.43)	.80	0.88 (0.53-1.41)	0.62	1.24 (0.45-3.01)	.66
Arterial access site (vs femoral)						
Radial	0.72 (0.54-0.96)	.025	0.79 (0.55-1.12)	0.19	0.56 (0.34-0.92)	.021
Others	1.18 (0.77-1.77)	.44	1.39 (0.85-2.21)	.18	0.77 (0.31-1.73)	.54
Three-vessel disease	1.34 (1.04-1.71)	.022	1.30 (0.95-1.76)	.093	1.33 (0.86-2.06)	.20
LMT or proximal LAD disease	0.92 (0.72-1.18)	.51	0.84 (0.62-1.14)	.26	0.98 (0.64-1.51)	.94
Drug-eluting stent use	0.81 (0.62-1.07)	.14	0.91 (0.65-1.29)	.60	0.70 (0.43-1.15)	.15
Mechanical circulatory support during PCI	5.03 (3.85-6.57)	<.001	5.59 (4.03-7.75)	<.001	4.18 (2.60-6.79)	<.001
Annual PCI volume tertile (vs low tertile)						
Middle	1.01 (0.72-1.44)	.94	0.94 (0.62-1.45)	.79	0.95 (0.50-1.86)	.88
High	0.90 (0.65-1.27)	.54	1.03 (0.70-1.56)	.88	0.58 (0.31-1.12)	.098

CABG, coronary artery bypass grafting; LAD, left anterior descending artery; LMT, left main trunk; NSTE-ACS, non–ST-segment elevation acute coronary syndrome; PCI, percutaneous coronary intervention; RA, rotational atherectomy, STEMI, ST-segment elevation myocardial infarction.

Adjusted odds ratios for any bleeding events requiring blood transfusion or any complications after RA are shown in Table 4. Advanced age, female, chronic kidney disease, three-vessel disease, and mechanical circulatory support during PCI were significantly associated with increased bleeding events after RA in patients with ACS (*P* < .05 for all). Similar to in-hospital mortality, the occurrence of any complications after RA was more frequent in patients with ACS with multiple comorbidities. These included advanced age, chronic kidney disease, prior coronary artery bypass grafting, cardiac arrest within 24 hours, cardiogenic shock within 24 hours, 3-vessel disease, and mechanical circulatory support during PCI (*P* < .05 for all).

**Table 4:** Adjusted odds ratios of any bleeding events that required blood transfusion or all complications after rotational atherectomy for acute coronary syndrome.

Variables	Any bleeding events		All complications	
	Odds ratio (95% CI)	*P* value	Odds ratio (95% CI)	*P* value
Age, per 1 y	1.04 (1.01-1.06)	.001	1.03 (1.02-1.04)	<.001
Female sex	1.77 (1.22-2.58)	.003	1.45 (1.21-1.75)	<.001
Hypertension	0.74 (0.48-1.17)	.18	0.86 (0.69-1.08)	.20
Diabetes	0.97 (0.68-1.39)	.88	1.20 (1.00-1.43)	.050
Hyperlipidemia	0.89 (0.62-1.29)	.53	0.87 (0.73-1.05)	.14
Current/recent smoker (within 1 year)	1.25 (0.78-1.94)	.34	1.10 (0.88-1.37)	.39
Chronic kidney disease	1.85 (1.26-2.71)	.002	1.45 (1.19-1.76)	<.001
Dialysis	0.68 (0.40-1.14)	.15	1.19 (0.92-1.52)	.18
Chronic lung disease	1.35 (0.55-2.81)	.47	1.14 (0.73-1.72)	.55
Peripheral arterial disease	0.85 (0.48-1.41)	.55	1.23 (0.97-1.56)	.090
Prior PCI	0.89 (0.58-1.35)	.59	0.84 (0.69-1.03)	.097
Prior CABG	1.04 (0.52-1.90)	.91	1.45 (1.09-1.92)	.009
Prior myocardial infarction	1.10 (0.70-1.71)	.67	1.03 (0.82-1.28)	.82
STEMI	1.10 (0.71-1.66)	.67	1.18 (0.95-1.46)	.13
Cardiac arrest within 24 hours	1.71 (0.89-3.19)	.096	2.59 (1.83-3.67)	<.001
Cardiogenic shock within 24 hours	1.30 (0.78-2.13)	.31	2.17 (1.69-2.79)	<.001
Preprocedural potent P2Y12 inhibitors (ticagrelor or prasugrel)	1.36 (0.96-1.95)	.090	1.12 (0.94-1.33)	.20
Preprocedural anticoagulants	1.04 (0.53-1.86)	.90	1.28 (0.95-1.71)	.10
Arterial access site (vs femoral)				
Radial	0.69 (0.46-1.03)	.072	0.85 (0.70-1.04)	.12
Others	1.51 (0.83-2.61)	.16	1.11 (0.80-1.51)	.53
Three-vessel disease	1.72 (1.20-2.45)	.003	1.34 (1.12-1.61)	.002
LMT or proximal LAD disease	1.26 (0.88-1.81)	.21	0.88 (0.73-1.05)	.14
Drug-eluting stent use	1.19 (0.77-1.92)	.44	0.91 (0.75-1.12)	.38
Mechanical circulatory support during PCI	3.50 (2.35-5.21)	<.001	5.87 (4.85-7.12)	<.001
Annual PCI volume tertile (vs low tertile)				
Middle	1.31 (0.77-2.31)	.34	1.03 (0.80-1.33)	.80
High	1.33 (0.80-2.30)	.29	0.94 (0.74-1.20)	.61

CABG, coronary artery bypass grafting; LAD, left anterior descending artery; LMT, left main trunk; PCI, percutaneous coronary intervention; STEMI, ST-segment elevation myocardial infarction.

Table 5 shows the adjusted odds ratios for in-hospital death in patients with ACS undergoing RA, including hospitals that began performing coronary atherectomy after the 2020 policy change as a covariate. RA performed at these newly accredited hospitals did not show a significant association with in-hospital mortality after RA in patients with ACS (adjusted odds ratio, 0.99; 95% CI, 0.63-1.51; *P* = .95).

**Table 5:** Adjusted in-hospital death when including hospitals launching coronary atherectomy after 2020 as a covariate.

Variables	All RA cases		RA for NSTE-ACS		RA for STEMI	
	Odds ratio (95% CI)	*P* value	Odds ratio (95% CI)	*P* value	Odds ratio (95% CI)	*P* value
Age, per 1 y	1.06 (1.04-1.08)	<.001	1.08 (1.06-1.10)	<.001	1.04 (1.01-1.06)	.005
Female sex	1.16 (0.89-1.50)	.265	1.18 (0.85-1.63)	.32	1.17 (0.74-1.86)	.51
Hypertension	0.79 (0.58-1.07)	.123	0.71 (0.49-1.04)	.069	0.98 (0.58-1.69)	.94
Diabetes	1.39 (1.09-1.80)	.009	1.30 (0.95-1.77)	.099	1.61 (1.04-2.51)	.034
Hyperlipidemia	0.60 (0.47-0.76)	<.001	0.53 (0.39-0.72)	<.001	0.81 (0.53-1.25)	.35
Current/recent smoker (within 1 y)	1.01 (0.74-1.37)	.93	1.10 (0.74-1.60)	.63	0.68 (0.39-1.16)	.17
Chronic kidney disease	1.52 (1.16-2.00)	.003	1.56 (1.11-2.21)	.012	1.48 (0.92-2.37)	.10
Dialysis	1.48 (1.07-2.05)	.017	1.81 (1.23-2.68)	.003	1.15 (0.60-2.18)	.67
Chronic lung disease	1.25 (0.70-2.11)	.43	1.36 (0.66-2.54)	.37	1.24 (0.42-3.17)	.67
Peripheral arterial disease	1.47 (1.07-2.00)	.015	1.89 (1.32-2.68)	<.001	0.73 (0.36-1.42)	.37
Prior PCI	0.70 (0.53-0.93)	.015	0.74 (0.53-1.05)	.091	0.85 (0.48-1.48)	.57
Prior CABG	1.78 (1.23-2.56)	.002	2.07 (1.36-3.10)	.001	1.88 (0.72-4.62)	.18
Prior myocardial infarction	1.19 (0.88-1.61)	.25	1.67 (1.18-2.37)	.004	0.48 (0.25-0.92)	.030
Cardiac arrest within 24 hours	3.58 (2.46-5.22)	<.001	3.42 (2.01-5.78)	<.001	3.35 (1.91-5.88)	<.001
Cardiogenic shock within 24 hours	3.47 (2.57-4.67)	<.001	3.60 (2.44-5.28)	<.001	2.54 (1.54-4.20)	<.001
Preprocedural potent P2Y12 inhibitors (ticagrelor or prasugrel)	1.22 (0.96-1.55)	.11	1.33 (0.99-1.80)	.057	0.90 (0.59-1.40)	.65
Preprocedural anticoagulants	0.90 (0.58-1.35)	.62	0.88 (0.53-1.40)	.60	1.22 (0.45-2.97)	.68
Arterial access site (vs femoral)						
Radial	0.72 (0.54-0.95)	.021	0.79 (0.55-1.13)	.20	0.56 (0.34-0.91)	.020
Others	1.17 (0.76-1.75)	.47	1.40 (0.85-2.23)	.17	0.77 (0.31-1.73)	.54
Three-vessel disease	1.32 (1.03-1.69)	.026	1.30 (0.96-1.76)	.091	1.33 (0.85-2.05)	.20
LMT or proximal LAD disease	0.89 (0.69-1.13)	.34	0.84 (0.62-1.14)	.26	0.98 (0.64-1.51)	.94
Drug-eluting stent use	0.80 (0.61-1.06)	.12	0.91 (0.65-1.29)	.59	0.71 (0.44-1.16)	.16
Mechanical circulatory support during PCI	5.31 (4.07-6.93)	<.001	5.58 (4.03-7.74)	<.001	4.18 (2.60-6.79)	<.001
Annual PCI volume tertile (vs low tertile)						
Middle	1.03 (0.72-1.50)	.87	0.99 (0.63-1.57)	.97	0.91 (0.47-1.82)	.79
High	0.94 (0.65-1.36)	.73	1.10 (0.70-1.74)	.69	0.55 (0.28-1.09)	.083
Hospitals launching coronary atherectomy after the device policy change in 2020	0.99 (0.63-1.51)	.95	1.17 (0.68-1.94)	.56	0.80 (0.33-1.79)	.60

CABG, coronary artery bypass grafting; LAD, left anterior descending artery; LMT, left main trunk; NSTE-ACS, non–ST-segment elevation acute coronary syndrome; PCI, percutaneous coronary intervention; RA, rotational atherectomy, STEMI, ST-segment elevation myocardial infarction.

### In-hospital outcomes after OA for ACS

Supplemental Table S1 presents the adjusted odds ratios for in-hospital death after coronary atherectomy, including OA as a covariate. The use of OA for ACS did not significantly differ from RA with respect to in-hospital mortality after PCI (adjusted odds ratio, 0.79; 95% CI, 0.34-1.62; *P* = .54), although a nonsignificant interaction was observed between OA use and STEMI (*P* = .073). When analyzing patients with STEMI specifically, OA use showed a significant decrease in in-hospital mortality after PCI for ACS compared to RA (adjusted odds ratio, 0.17; 95% CI, 0.025-0.73; *P* = .034).

## Discussion

Several important findings of this study are as follows (Central Illustration): (1) the overall rate of PCI with coronary atherectomy for ACS was low in Japan; however, RA for NSTE-ACS was increasingly performed between 2019 and 2022; (2) there was significant variation in the rate of RA for ACS among hospitals in Japan; (3) increased complexity of baseline characteristics was significantly correlated with increased rates of in-hospital mortality, any bleeding events, and complications after RA for ACS; (4) the transradial approach was significantly associated with a lower odds ratio of in-hospital death after RA for ACS than the transfemoral approach; and (5) annual hospital PCI volume and hospitals implementing coronary atherectomy after the 2020 policy change in Japan did not show significant associations with in-hospital outcomes after RA for ACS. Overall, the present study represents the largest analysis to date focusing on the trends and outcomes of coronary atherectomy for ACS in Japan.

**Central Illustration:**
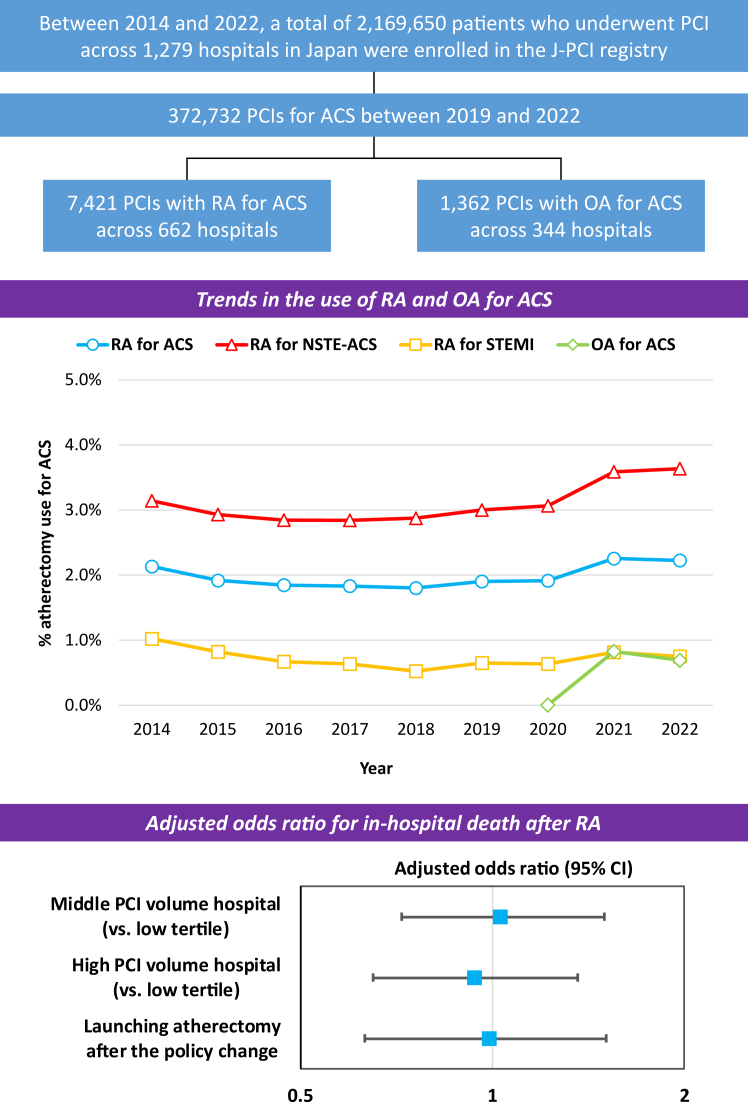
This study aimed to investigate the temporal trends and hospital variability in utilization and outcomes of coronary atherectomy for acute coronary syndrome (ACS) concerning the policy change regarding coronary atherectomy, using a nationwide percutaneous coronary intervention (PCI) registry in Japan. The study found that the overall rate of PCI with coronary atherectomy for ACS was low in Japan; however, rotational atherectomy (RA) for non–ST-segment elevation (NSTE)-ACS was increasingly performed between 2019 and 2022. Forest plots of the adjusted odds ratio for in-hospital death after RA for ACS show that annual hospital PCI volume and hospitals launching coronary atherectomy after the 2020 policy change in Japan did not show significant associations with in-hospital outcomes after RA for ACS. The bars show 95% confidence intervals (CIs) of the odds ratio. OA, orbital atherectomy; STEMI, ST-segment elevation myocardial infarction.

We observed a low rate of PCI with coronary atherectomy for ACS in contemporary practice; however, a trend toward increasing the use of RA for NSTE-ACS was observed. In addition, a significant variation in the rate of RA for ACS was observed across hospitals (46% of hospitals did not perform RA for ACS between 2014 and 2022). The overall utilization rate of coronary atherectomy devices for both chronic coronary syndrome and ACS ranges from 1% to 3% in Europe,^3^ US,^7^ and Japan.^8^^,^^23^ The low utilization rate of coronary atherectomy for ACS in this study is consistent with that observed for all PCI procedures. RA for NSTE-ACS was increasingly performed between 2019 and 2022, likely owing to the 2020 policy change on coronary atherectomy in Japan. Coronary atherectomy has unique complications, including slow flow, coronary perforation, and entrapment of the atherectomy burr or crown.^2^ Although these complications can lead to cardiac tamponade, emergent surgery, and death, a previous report from the J-PCI registry showed the incidence of these important complications after RA was 1.3%, with emergent surgery required in 0.18% of cases.^8^ Consequently, from April 2020, PCI operators in Japan were permitted to use coronary atherectomy devices without on-site surgical backup, resulting in an increase in procedures performed at low-volume PCI hospitals or at newly accredited hospitals that began performing coronary atherectomy without on-site surgical backup after the policy change.

The increasing complexity of baseline characteristics was associated with increased in-hospital mortality, bleeding events, and complications after RA for ACS. However, hospital PCI volume and newly accredited hospitals starting coronary atherectomy after the policy change were not associated with in-hospital outcomes after RA for ACS. The inverse relationship between hospital PCI volume and outcomes after PCI has been reported in various PCI cohorts.^19^^,^^24^^,^^25^ Sakakura et al,^8^ using data from the J-PCI registry between 2013 and 2014, reported that patients treated with RA at high-volume hospitals (≥25 PCI with RA/2 years) had a lower rate of in-hospital complications than those at low-volume hospitals (≤10 PCI with RA/2 years: adjusted odds ratio, 0.56; 95% CI, 0.36-0.89; *P* = .011 for high-volume vs low-volume hospitals). Beohar et al,^7^ using data from the National Cardiovascular Data Registry CathPCI registry in the US between 2009 and 2016, reported that increasing quartile of hospital annual coronary atherectomy volume was associated with lower in-hospital mortality (adjusted odds ratio, 0.85; 95% CI, 0.76-0.96; *P* < .001). Several possible explanations exist for the nonsignificant relationship between hospital PCI volume and in-hospital outcomes in the present study. First, the low event rates in this study may have resulted in insufficient power to detect significant differences between low- and high-volume hospitals. Second, treatment strategies for severe coronary calcification have been standardized over the last decade to prevent complications following coronary atherectomy, such as intravascular imaging assessment of calcified lesions, burr size (proposed consensus for maximal RA burr-to-artery ratio of ≤0.6), minimizing sheath size depending on burr size requirement, and rotational speed.^1^, ^2^, ^3^, ^4^ Furthermore, since 2020, device-specific training on coronary atherectomy has been mandatory for low RA volume hospitals (≤10 PCI with RA/2 years) in Japan. These recent advances in coronary atherectomy strategy and device-specific training may have mitigated the differences between high- and low-volume hospitals.

The transradial approach was significantly associated with a lower odds ratio of in-hospital death after RA for ACS than the transfemoral approach. This finding aligns with previous studies investigating in-hospital outcomes after PCI for various indications^19^^,^^26^^,^^27^ and comorbidities.^20^ Although sheath size depends on burr size requirements, a 6F PCI system is sufficient to support OA and RA with ≤1.75 mm burr.^4^ Considering that the initial goal of PCI for ACS is to achieve thrombolysis in myocardial infarction 3 flow, using coronary atherectomy via the transradial approach with a smaller burr size or slender introducer sheath may be a reasonable choice even in the setting of ACS.

Notably, the use of drug-eluting stents was common but decreased from 79.7% in 2019 to 73.0% in 2022, whereas the use of drug-coated balloons increased from 21.3% in 2019 to 32.2% in 2022. This shift may be caused by recent advances in stentless PCI for de novo lesions using drug-coated balloons.^28^^,^^29^ Although coronary atherectomy for severely calcified lesions is performed to enhance the stent delivery and expansion, stent malapposition at calcified nodules is frequently observed even after lesion modification by coronary atherectomy.^30^ Given that the best management strategy for severely calcified lesions in patients with ACS is still debatable, a “leave nothing behind” approach using a drug-coated balloon may be an attractive option in patients undergoing coronary atherectomy^31^; however, this requires further investigation. RA and OA can also be used for stent ablation in restenotic lesions caused by underexpanded stents. Because of the increased risk of burr entrapment during stent ablation, on-site surgical backup may be required in the ACS setting.^2^ Coronary lithoplasty may be another option to resolve stent underexpansion due to severe calcification at hospitals without on-site surgical backup.^32^

Our exploratory analysis suggested that OA might be more effective than RA in reducing mortality among patients with STEMI undergoing coronary atherectomy. Although OA and RA have comparably favorable outcomes after PCI in the literature,^33^ another propensity score–matched study reported that procedure-related myocardial infarction occurred less frequently with OA compared to RA (6.7% vs 13.8%, *P* < .01).^34^ Furthermore, a recent randomized trial on coronary atherectomy in patients with chronic coronary syndrome revealed that the maximum atherectomy area assessed by optical coherence tomography was significantly larger in the RA group than in the OA group (1.34 [IQR, 1.02-1.89] mm^2^ vs 0.83 [IQR, 0.59-1.11] mm^2^; *P* = .004).^35^ This suggests that the larger atherectomy area created by RA may have a negative effect on thrombotic lesions of STEMI compared to OA.

### Study limitations

The present study had several limitations. The J-PCI registry is a large nationwide multicenter registry collecting clinical, procedural, and institutional data elements at participating centers via a web-based interface. However, culprit lesion morphology and procedural details including lesion length, severity of calcification, presence or absence of thrombus, SYNTAX score,^36^ stent type, the occurrence of slow flow or no reflow during coronary atherectomy, and use of intravascular imaging were not available. Nevertheless, intravascular imaging is used more frequently in Japan (85%-90% of all PCI) compared to Europe and other regions.^37^, ^38^, ^39^ The lack of data on culprit lesion morphology precluded an exploratory comparison of in-hospital outcomes in patients with ACS undergoing PCI with or without coronary atherectomy, as a propensity score model without these data cannot fully exclude selection bias in the use of coronary atherectomy devices. The small sample size of patients undergoing OA also limited an adequate comparison between RA and OA for ACS. Finally, long-term data were not available in this study; therefore, further studies are needed to verify the validity of the policy change on coronary atherectomy in Japan.

## Conclusion

The present study using a nationwide multicenter PCI registry showed that the overall rate of coronary atherectomy for ACS was low even after the 2020 policy change on coronary atherectomy in Japan. Hospital PCI volume and on-site surgical backup were not significantly associated with in-hospital mortality after coronary atherectomy for ACS. Our data suggest that coronary atherectomy for ACS may be feasible even in hospitals with low PCI volume without on-site surgical backup.

## Declaration of competing interest

Yuya Matsue received honoraria from Otsuka Pharmaceutical Co, Novartis Pharma K.K., Bayer Inc, and AstraZeneca, and research grants from Pfizer Japan Inc, Otsuka Pharmaceutical Co, EN Otsuka Pharmaceutical Co, Ltd, and Nippon Boehringer Ingelheim Co, Ltd. Ken Kozuma received honoraria from Boston Scientific, Abbott Medical, Medtronic, Otsuka, Daiichi-Sankyo, Amgen, Novartis, Boehringer, Bayer, Life Science Institute, Mochida, and Novo Nordisk Pharma; and received Scholarship fund from Abbott Medical. The other authors have no conflicts of interest to declare.

## Funding sources

This work was supported in part by JSPS KAKENHI Grant Number 22K15867 (Grant-in-Aid for Early-Career Scientists to Tadao Aikawa) and Sakakibara Memorial Research Grant from the Sakakibara Heart Foundation (to Tadao Aikawa).

## Ethics statement and patient consent

The study protocol of the J-PCI registry was approved by the Institutional Review Board Committee of the Network for Promotion of Clinical Studies (a specified nonprofit organization affiliated with Osaka University Graduate School of Medicine [Osaka, Japan]) in compliance with the Declaration of Helsinki. Written informed consent was waived because of the retrospective and observational nature of the study.

## Footnotes

To access the supplementary material accompanying this article, visit the online version of the *Journal of the Society for Cardiovascular Angiography & Interventions* at 10.1016/j.jscai.2025.103622.
