# Supplementary material for: Application and Outcomes of Coronary Atherectomy in Acute Coronary Syndrome: A Report From the J-PCI Registry
Source: J Soc Cardiovasc Angiogr Interv. 2025 May 13;4(7):103622. doi: 10.1016/j.jscai.2025.103622 (PMC12418442; doi:10.1016/j.jscai.2025.103622)
Supplement: Supplementary Material [file mmc1.docx]

**SUPPLEMENTARY METHODS**

**Definitions of categories on acute coronary syndrome**

Unstable angina

At least one of the following is met:

1) New-onset angina: angina, which manifested within the past month.

2) Increasing angina: angina that worsened within the past month.

3) Resting angina: persistent angina at rest or angina that markedly restricts daily life (symptoms triggered by walking tens of meters or one flight of stairs).

4) Postinfarction angina: persistent angina within 1 month following a myocardial infarction event with the involvement of elevated ST segments on ECG or cardiac biomarkers; if they are, the angina is defined as STEMI or NSTEMI, respectively.

Acute myocardial infarction

Persistent myocardial ischemia symptoms accompanied by elevated cardiac markers. Elevated cardiac biomarkers refer to elevated creatine kinase (CK) or CK-MB levels [two-folds higher than the normal values] or elevated troponin levels [≥ 99th percentile].

Acute myocardial infarctions are classified as STEMI or NSTEMI as described below:

1) ST-elevation myocardial infarction (STEMI): ST elevation on two or more contiguous leads (≥ 0.2 mV in a precordial lead at the J point or ≥ 0.1 mV in a limb lead), new left bundle branch block, or posterior myocardial infarction on a 12-lead ECG.

2) Non-ST-elevation myocardial infarction (NSTEMI): ECG changes either do not qualify as ST elevation or are not present at all.

**Supplemental Figure S1.** Trends in the use of drug-eluting stent (DES) or drug-coated balloon after rotational atherectomy (RA) for acute coronary syndrome (ACS) based on numbers (**A**) and percentages (**B**) of PCI cases.

**A**

**
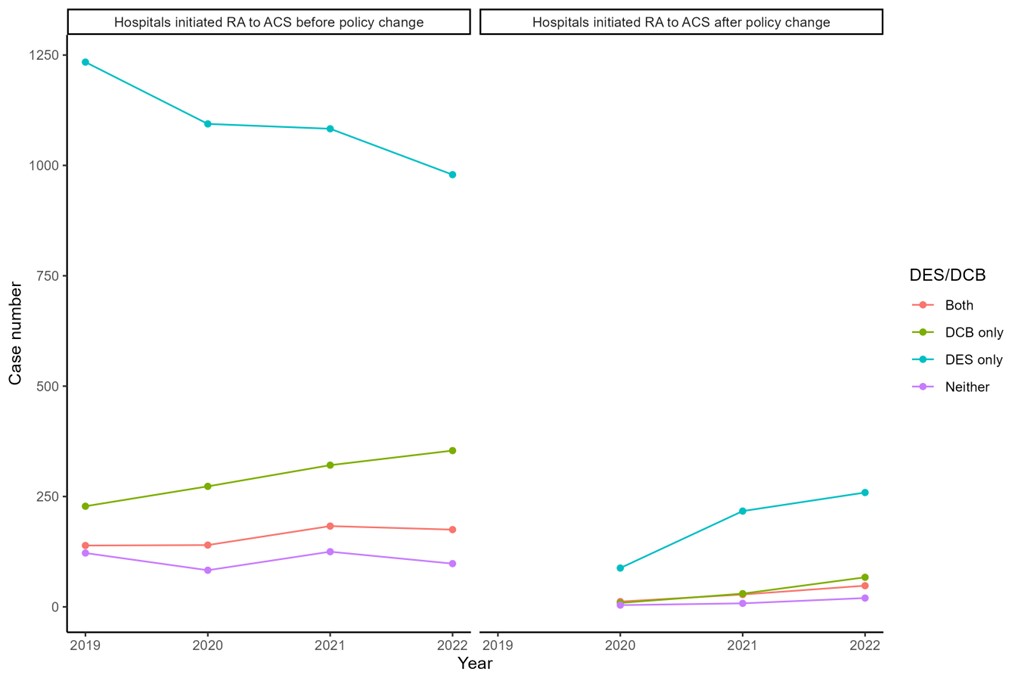
**

**B**

**
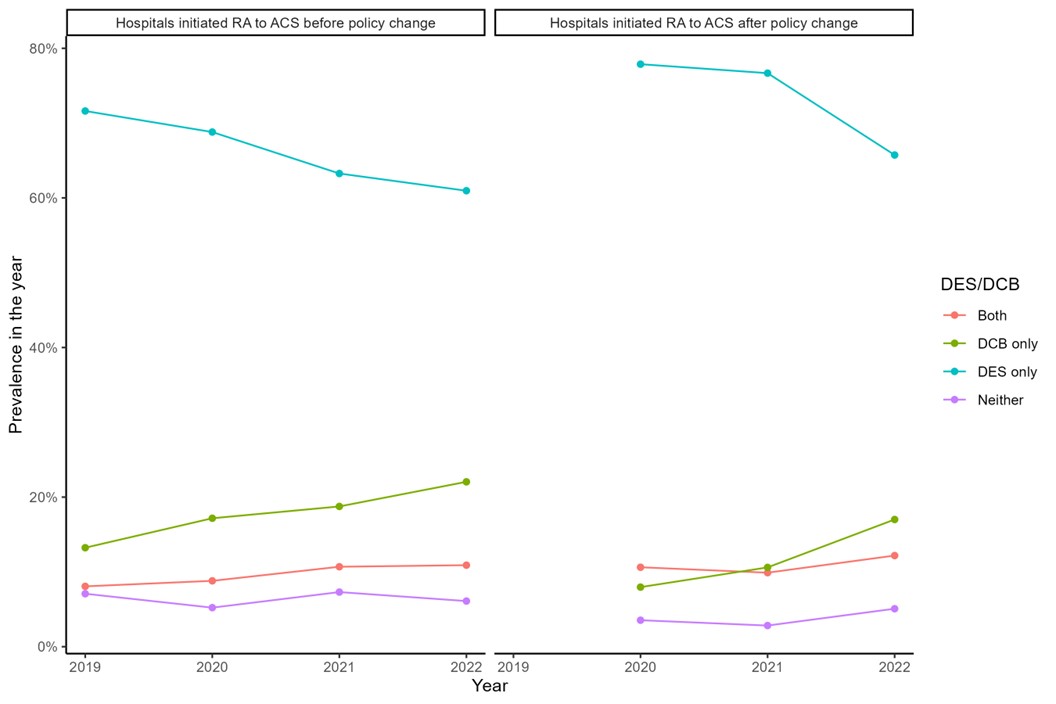
**

**Supplemental Table S1.** Adjusted odds ratios of in-hospital death when including orbital atherectomy as a covariate.

|  | **All atherectomy cases** |  | **Atherectomy for NSTE-ACS** |  | **Atherectomy for STEMI** |  |
| --- | --- | --- | --- | --- | --- | --- |
| **Variables** | **Odds ratio**  **(95% CI)** | **P value** | **Odds ratio**  **(95% CI)** | **P value** | **Odds ratio**  **(95% CI)** | **P value** |
| Age, per 1 y | 1.06 (1.05-1.08) | <0.001 | 1.08 (1.06-1.10) | <0.001 | 1.04 (1.01-1.06) | 0.003 |
| Female patients | 1.13 (0.87-1.47) | 0.36 | 1.18 (0.85-1.63) | 0.31 | 1.19 (0.74-1.88) | 0.47 |
| Hypertension | 0.79 (0.59-1.08) | 0.13 | 0.70 (0.49-1.04) | 0.068 | 0.98 (0.58-1.69) | 0.94 |
| Diabetes | 1.43 (1.11-1.85) | 0.006 | 1.30 (0.96-1.78) | 0.098 | 1.62 (1.05-2.54) | 0.032 |
| Hyperlipidemia | 0.61 (0.47-0.78) | <0.001 | 0.53 (0.39-0.72) | <0.001 | 0.81 (0.52-1.24) | 0.33 |
| Current/recent smoker (within 1 y) | 0.98 (0.71-1.33) | 0.89 | 1.10 (0.74-1.60) | 0.63 | 0.68 (0.39-1.16) | 0.17 |
| Chronic kidney disease | 1.54 (1.17-2.03) | 0.002 | 1.57 (1.11-2.22) | 0.011 | 1.46 (0.91-2.34) | 0.12 |
| Dialysis | 1.58 (1.13-2.19) | 0.007 | 1.82 (1.23-2.69) | 0.003 | 1.17 (0.61-2.22) | 0.63 |
| Chronic lung disease | 1.23 (0.68-2.09) | 0.47 | 1.35 (0.66-2.54) | 0.37 | 1.22 (0.41-3.11) | 0.70 |
| Peripheral arterial disease | 1.49 (1.09-2.03) | 0.012 | 1.88 (1.31-2.67) | <0.001 | 0.71 (0.35-1.39) | 0.33 |
| Prior PCI | 0.74 (0.55-0.98) | 0.038 | 0.75 (0.53-1.05) | 0.099 | 0.88 (0.49-1.53) | 0.65 |
| Prior CABG | 1.89 (1.30-2.73) | 0.001 | 2.08 (1.36-3.12) | 0.001 | 1.80 (0.69-4.48) | 0.22 |
| Prior myocardial infarction | 1.23 (0.90-1.66) | 0.19 | 1.66 (1.17-2.35) | 0.005 | 0.46 (0.23-0.88) | 0.022 |
| STEMI | 1.97 (1.50-2.58) | <0.001 | - | - | - | - |
| Cardiac arrest within 24 h | 3.48 (2.38-5.08) | <0.001 | 3.46 (2.04-5.84) | <0.001 | 3.49 (1.99-6.15) | <0.001 |
| Cardiogenic shock within 24 h | 3.13 (2.31-4.24) | <0.001 | 3.57 (2.42-5.24) | <0.001 | 2.60 (1.57-4.29) | <0.001 |
| Pre-procedural potent P2Y12 inhibitors (ticagrelor or prasugrel) | 1.20 (0.95-1.53) | 0.13 | 1.34 (1.00-1.81) | 0.050 | 0.93 (0.60-1.43) | 0.73 |
| Pre-procedural anticoagulants | 0.95 (0.61-1.44) | 0.83 | 0.88 (0.53-1.41) | 0.62 | 1.36 (0.49-3.34) | 0.53 |
| Arterial access site (vs. femoral) |  |  |  |  |  |  |
| Radial | 0.71 (0.54-0.95) | 0.021 | 0.79 (0.55-1.13) | 0.20 | 0.55 (0.33-0.89) | 0.016 |
| Others | 1.17 (0.76-1.76) | 0.46 | 1.38 (0.84-2.20) | 0.19 | 0.75 (0.31-1.71) | 0.52 |
| Three-vessel disease | 1.34 (1.04-1.71) | 0.021 | 1.30 (0.95-1.76) | 0.095 | 1.34 (0.86-2.08) | 0.19 |
| LMT or proximal LAD disease | 0.91 (0.71-1.16) | 0.44 | 0.84 (0.62-1.14) | 0.27 | 0.95 (0.62-1.46) | 0.81 |
| Drug-eluting stent use | 0.81 (0.62-1.08) | 0.14 | 0.91 (0.65-1.29) | 0.58 | 0.69 (0.43-1.14) | 0.14 |
| Mechanical circulatory support during PCI | 5.09 (3.90-6.66) | <0.001 | 5.61 (4.05-7.78) | <0.001 | 4.27 (2.65-6.94) | <0.001 |
| Annual PCI volume tertile  (vs. low tertile) |  |  |  |  |  |  |
| Middle | 1.02 (0.72-1.45) | 0.92 | 0.94 (0.62-1.44) | 0.76 | 0.98 (0.51-1.92) | 0.95 |
| High | 0.92 (0.66-1.30) | 0.63 | 1.03 (0.70-1.56) | 0.87 | 0.62 (0.33-1.20) | 0.14 |
| Orbital atherectomy (vs. only RA) | 0.79 (0.34-1.62) | 0.54 | 0.76 (0.32-1.59) | 0.50 | 0.17 (0.02-0.73) | 0.034 |
| Interaction (orbital atherectomy × STEMI) | 0.20 (0.03-1.02) | 0.073 | - | - | - | - |

CABG, coronary artery bypass grafting; CI, confidence interval; LAD, left anterior descending artery; LMT, left main trunk; NSTE-ACS, non-ST-segment elevation acute coronary syndrome; PCI, percutaneous coronary intervention; RA, rotational atherectomy, STEMI, ST-segment elevation myocardial infarction.
